# The impact of atmospheric rivers on rainfall in New Zealand

**DOI:** 10.1038/s41598-021-85297-0

**Published:** 2021-03-12

**Authors:** Jingxiang Shu, Asaad Y. Shamseldin, Evan Weller

**Affiliations:** 1 Department of Civil and Environmental Engineering, The University of Auckland, Auckland Mail Centre, Private Bag 92019, Auckland, 1142 New Zealand; 2 School of Environment, The University of Auckland, Auckland Mail Centre, Private Bag 92019, Auckland, 1142 New Zealand

**Keywords:** Atmospheric science, Hydrology, Hydrology, Hydrology

## Abstract

This study quantifies the impact of atmospheric rivers (ARs) on rainfall in New Zealand. Using an automated AR detection algorithm, daily rainfall records from 654 rain gauges, and various atmospheric reanalysis datasets, we investigate the climatology of ARs, the characteristics of landfalling ARs, the contribution of ARs to annual and seasonal rainfall totals, and extreme rainfall events between 1979 and 2018 across the country. Results indicate that these filamentary synoptic features play an essential role in regional water resources and are responsible for many extreme rainfall events on the western side of mountainous areas and northern New Zealand. In these regions, depending on the season, 40–86% of the rainfall totals and 50–98% of extreme rainfall events are shown to be associated with ARs, with the largest contributions predominantly occurring during the austral summer. Furthermore, the median daily rainfall associated with ARs is 2–3 times than that associated with other storms. The results of this study extend the knowledge on the critical roles of ARs on hydrology and highlight the need for further investigation on the landfalling AR physical processes in relation to global circulation features and AR sources, and hydrological hazards caused by ARs in New Zealand.

## Introduction

Atmospheric rivers (ARs) are global phenomena primarily occurring in the midlatitudes, distinguished by filamentary regions of strong horizontal water vapour transport in the atmosphere^1–7^. Firstly described by Newell et al.^2^, ARs are synoptic-scale filaments representing the 4 or 5 largest water vapour fluxes that occur between the midlatitudes and the tropics on any given day, and the length of an AR is generally five times its width. ARs often linked to extratropical cyclones and fronts^4,5^, the key mechanisms of precipitation^8,9^ and play a critical role in extreme precipitation events^10^ in the midlatitudes. As part of extratropical cyclones and fronts, ARs have significant hydrological impacts when making landfall for most mid-latitude regions globally, including extreme precipitation events^11–15^ and the frequency of the occurrence of droughts and floods^16^.

ARs significantly contribute to precipitation totals and produce heavy orographic precipitation when passing mountainous regions in the midlatitudes. Notable contributions of ARs to precipitation totals have been documented on the west coast of North America^17,18^, the central United States^19^, western Europe^20^, southern south America^21^, and South Africa^22^. ARs can contribute to more than 50% of annual or seasonal precipitation totals (cool season in particular) for these regions. Further, there is a strong connection between the heavy precipitation events and ARs in these regions^21–25^. For example, storm events (3-day precipitation total exceeds 400 mm) in California between the 1998 and 2008 water years are shown to be all associated with ARs^17^.

Rainfall caused by the combined effect of extratropical cyclones and their associated fronts with orographic lifting is typical in New Zealand^19^. Generally, this combined effect determines the location and intensity of heavy rainfall events across the country, commonly on the western side of mountainous areas and northern New Zealand^19,26^. For the South Island of the country, orographic precipitation is the major precipitation mechanism^27^. The highest annual precipitation totals in New Zealand (over 10,000 mm) occur in the Southern Alps^28^ as this extended mountain range provides a significant barrier to the prevailing airflows^29,30^ all year round. For the North Island, frontal systems and extratropical cyclones are the dominant precipitation mechanisms^19,31,32^, yet mountain ranges and volcanic mountain peaks in the North Island also provide obstacles to the prevailing airflows^19^.

Because of its geographic location, New Zealand’s climate is governed by two dynamical global circulation features^19^: the westerly wind belt^33–35^ to the south and the band of subtropical anticyclones^19^ to the north. As such, the seasonality of these global circulation features strongly influence the rainfall seasonality in the country^26,31,33,34,36,37^. For example, the overall seasonal pattern of rainfall in the North Island sees higher rainfall in winter (JJA) due to the increased occurrence of frontal systems when the country experiences more westerlies, and lower rainfall in summer (DJF) as the belt move towards the south^26,31,37^. Conversely, the South Island sees the opposite rainfall seasonal pattern^19^.

For New Zealand, several global AR studies have noted a considerable occurrence of landfalling ARs and their association with heavy precipitation events^14,15,38^ and the frequency of drought and flood occurrences^16^. However, these global studies are unable to reflect regions highly impacted by ARs, especially considering the diverse orography in New Zealand. Additionally, AR frequency and strength is projected to increase by 60% and 20%, respectively, under the most severe future climate scenario in the southern midlatitudes^39^. To this end, the hydrological impact of ARs is likely to be altered in the country. Therefore, evaluating the impact of ARs on rainfall at a local scale in New Zealand is essential, given the local climate features, the critical role that ARs play in the global water cycle and midlatitudes climate and hydrology, and the changing climate.

In this study, we hypothesise that this evaluation will be useful for better understanding the causes of both the spatial distribution of annual and seasonal rainfall totals and heavy rainfall events across the country to provide more useful information for better assessment of climate change impacts on hydrology. The objectives of the present study are (1) to investigate the climatology of ARs in New Zealand; (2) to evaluate the fraction of seasonal and annual rainfall totals linked with ARs; (3) to compare the intensity of AR rainfall and non-AR rainfall, and to investigate the association between the ARs and heavy rainfall events.

Briefly, ARs are defined as regions where the intensity of integrated water vapour transport (IVT) exceeds predefined thresholds with a length greater than 2,000 km and length/width ratio greater than 2^6,23,38^. An automated AR detection algorithm and six 6-hourly reanalysis datasets (Table S1) with varying spatial resolutions (ERA-interim: 0.125° × 0.125° and 1.5° × 1.5°, ERA-5: 0.25° × 0.25°, CFSR: 0.5° × 0.5° and 1.5° × 1.5°, MERRA-2: 0.5° × 0.625° during the period of 1979–2018, 1979–2018, 1979–2015, 1980–2019, respectively) were used to identify ARs around New Zealand. Detected landfalling ARs from each dataset were used to compare the characteristics of landfalling ARs and provide a general view of landfalling ARs in the country.

The assessment of AR impact on rainfall totals and daily rainfall intensity was based on daily rainfall records from 654 rain gauges with a mean length of 19 years from 1979 to 2018. Rain gauge locations are shown in Fig. 5. If an AR event coincides with a rainfall event (> 0 mm) within the same day at a rain gauge site, that day was labelled a “wet” AR day for that site, and that rain was considered to be associated with the AR. Note that ERA-interim ARs (with 0.125° × 0.125° spatial resolution) were used to assess the AR impact on rainfall in this study as using higher-resolution for this type of AR impact assessment whenever possible is recommended^25^. A more detailed description of the datasets, AR detection method, and methods to quantify ARs' impact on rainfall totals and daily rainfall intensity for each 654 rain gauge is provided in the Methods section.

## Results

### Climatology of ARs over New Zealand

This section presents the climatology of ERA-interim ARs and characteristics of the detected landfalling ARs at a 0.125° × 0.125° horizontal grid resolution over New Zealand from 1979 to 2018. Results from other databases are shown in the supplementary materials, where the spatial resolution of the other datasets examined is lower than that of ERA-interim. Note that the analysis in this section only represents ARs at some moment of their life cycle as the temporal resolution of all reanalysis datasets is 6 h.

**Figure 1:**
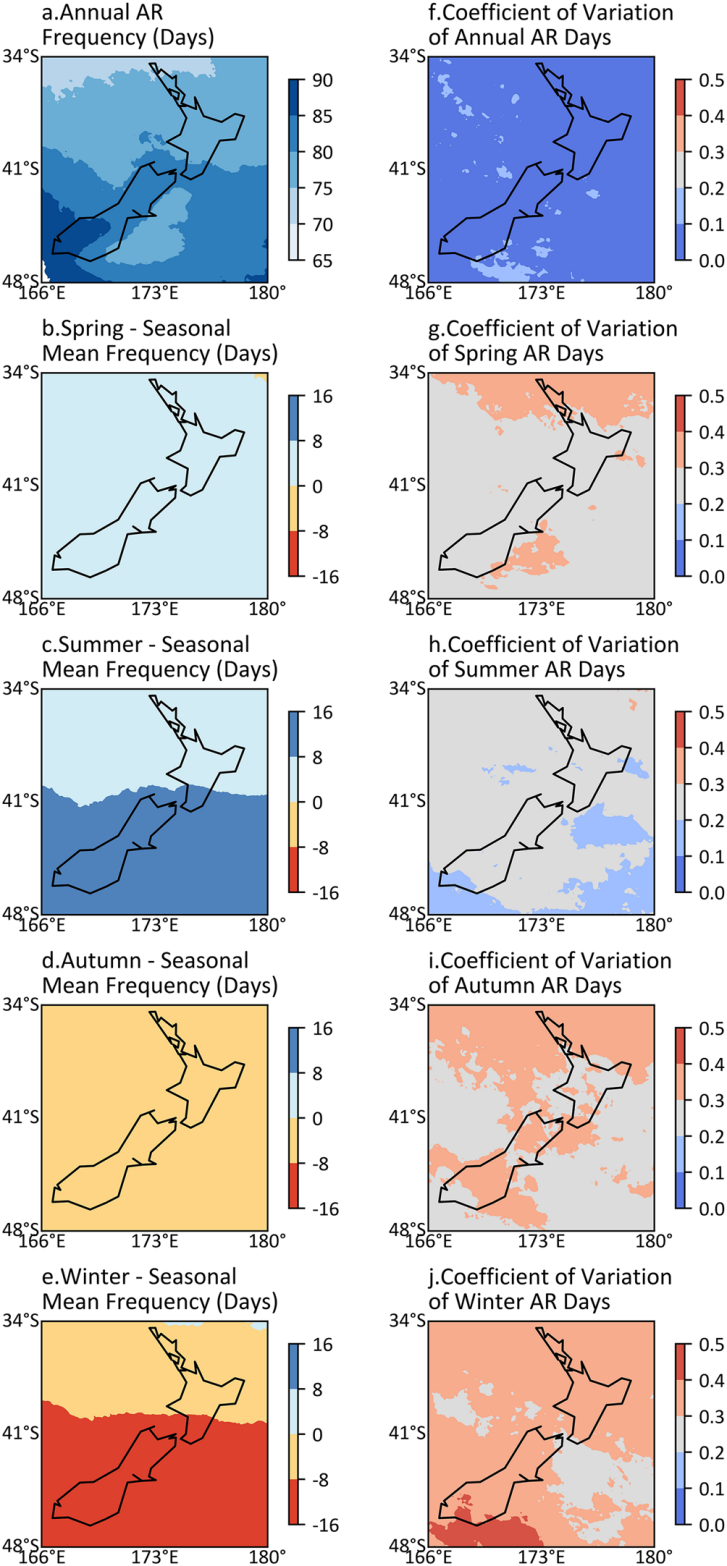
The climatology of AR frequency over New Zealand. (**a**) Annual frequency of AR days on each grid cell per year. (**b**–**e**) Difference between seasonal AR frequency and its seasonal mean frequency on each grid cell per year. (**f**–**j**) Annual and seasonal coefficients of variation of AR frequency for each grid cell. Note that months of seasons are: Spring (SON), Summer (DJF), Autumn (MAM), Winter (JJA).

The frequency of AR days and the coefficient of variation (CV) of AR days for each grid cell are shown in Fig. 1. Generally, there are 70 to 90 days per year with AR occurrence across New Zealand, and the South Island experiences more AR days than the North Island (Fig. 1a). The frequency of AR days is higher in warm seasons than in cool seasons, and the South Island experiences more AR days than the North Island in summer, yet fewer AR days in winter (Fig. 1b–e). The interannual variation for seasonal AR frequency (0.2–0.4) is notably higher than the annual scale (generally less than 0.1), and cool seasons experience larger variation compared with warm seasons (Fig. 1f–j). Coarsening the ERA-Interim and CFSR spatial resolution from 0.125° × 0.125° to 1.5° × 1.5° and 0.5° × 0.5° to 1.5° × 1.5°, respectively, results in slight differences in AR frequency and the CV of AR frequency over New Zealand. However, the seasonal pattern of AR frequency remains similar (see Figs. S1–S3).

**Figure 2:**
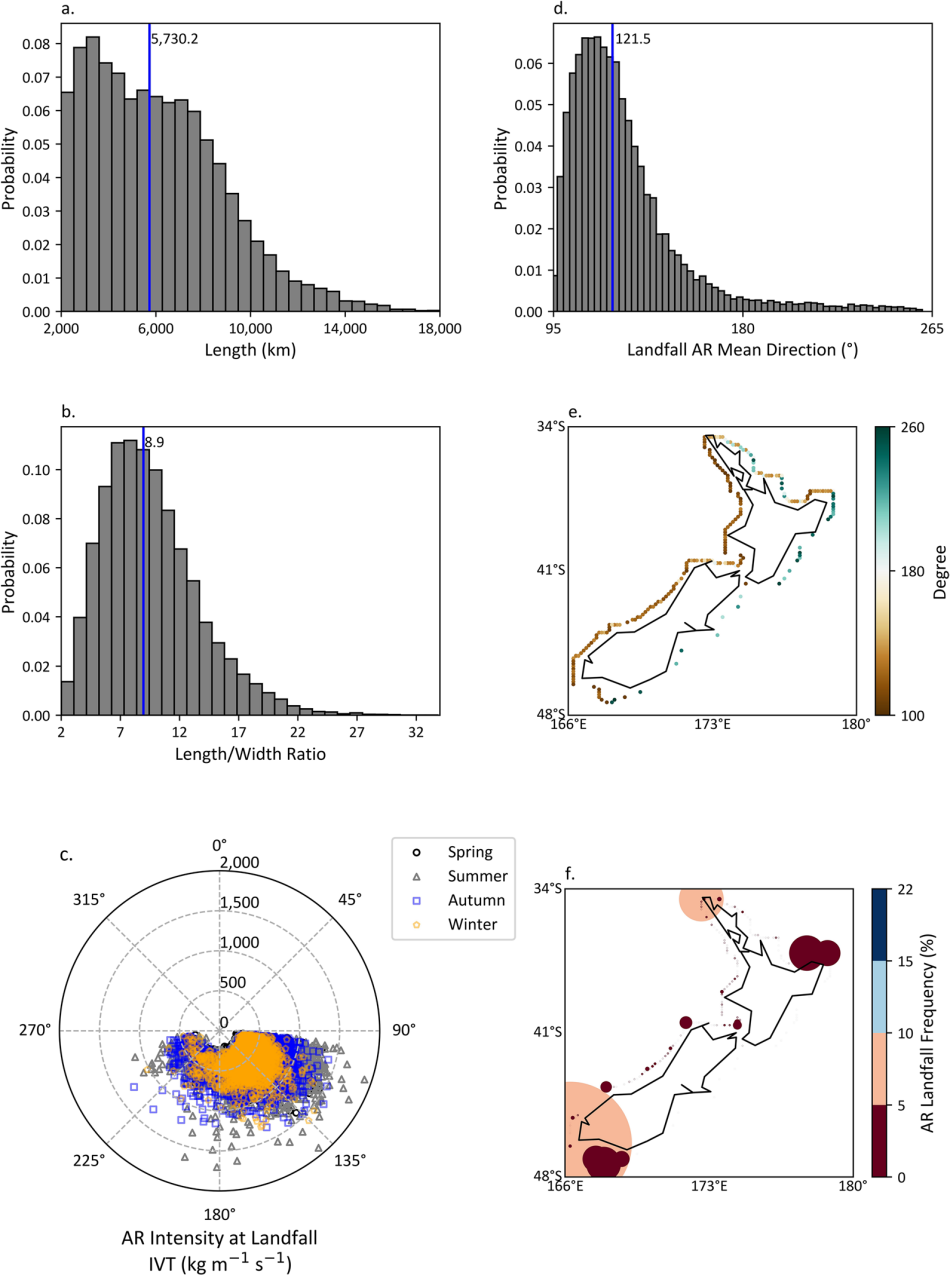
Histograms of detected landfalling ARs’ length (**a**), Length/width ratio (**b**), Landfalling AR mean direction (**d**), where blue vertical lines indicate the median value in each histogram. (**c**) Mean landfalling AR direction (angular coordinate) and seasonal landfalling AR intensity (radial coordinate) at landfall. (**e**) Landfalling ARs mean direction at landfall. (**f**) Proportional map of AR landfall frequency at landfall. Note that months of seasons are: Spring (SON), Summer (DJF), Autumn (MAM), Winter (JJA).

Geometric characteristics, mean landfalling AR direction and AR landfall frequency, and seasonal landfalling AR intensity and mean landfalling AR direction are shown in Fig. 2 (ERA-Interim) and Figs. S4–S6 (ERA-5, CFSR, and MERRA-2, respectively). The number of detected landfalling ARs and the mean percentage of monthly landfalling AR frequency is shown in Tables S1 and S2, respectively. The statistical characteristics of the landfalling AR length, mean direction, and length/width ratio generally agree with each other among the datasets, and all histograms are positively skewed (Figs. 2a,b,d and S4–S6a,b,d). Overall, the long and narrow feature of landfalling ARs is notable, the median of the length and the length/width ratio is about 5730 km (over 4700 km) and 8.9 (~ 8.4) for ERA-Interim (Fig. 2a,b) and other datasets (Figs. S4–S6a,b), respectively. In some cases, ARs can be longer than 10,000 km with a length/width ratio greater than 20. Coarsening the ERA-Interim and CFSR spatial resolution from 0.125° × 0.125° to 1.5° × 1.5° and 0.5° × 0.5° to 1.5° × 1.5° results in less landfalling ARs. For example, the number of detected landfalling ARs reduced from 17,876 to 4,057 and 12,904 to 3445, respectively (Table S1). Furthermore, datasets with coarser grid cells tend to reduce the occurrence of detected landfalling ARs regardless of the reanalysis dataset (Table S1).

Landfalling ARs commonly have a northwest direction, the median of the mean direction is about 121.5° (~ 123°) for ERA-Interim (Fig. 2d) and other datasets (Figs. S4–S6d). Moreover, ARs commonly approach the west side of New Zealand from a northwest direction, and in some cases from a northeast direction on the east side (Figs. 2e and S4–S6e). Summer landfalling ARs are generally stronger than those in winter (Figs. 2c and S4–S6c). The frequency of landfall along the west coasts is notable throughout the country, especially central-eastern and northern parts of the North Island, and southern parts of the South Island, with only small differences over southern parts of the North Island among the four datasets (Figs. 2f and S4–S6f). The seasonal pattern of landfalling AR occurrence is slightly different among the six datasets. However, the mean percentage of the occurrence in summer (30%) and spring (29%) is greater than in autumn (23%) and winter (20%), and the differences for the mean percentage of the monthly occurrences are all less than 3% (Table S2).

### Contribution of ARs to rainfall totals

**Figure 3:**
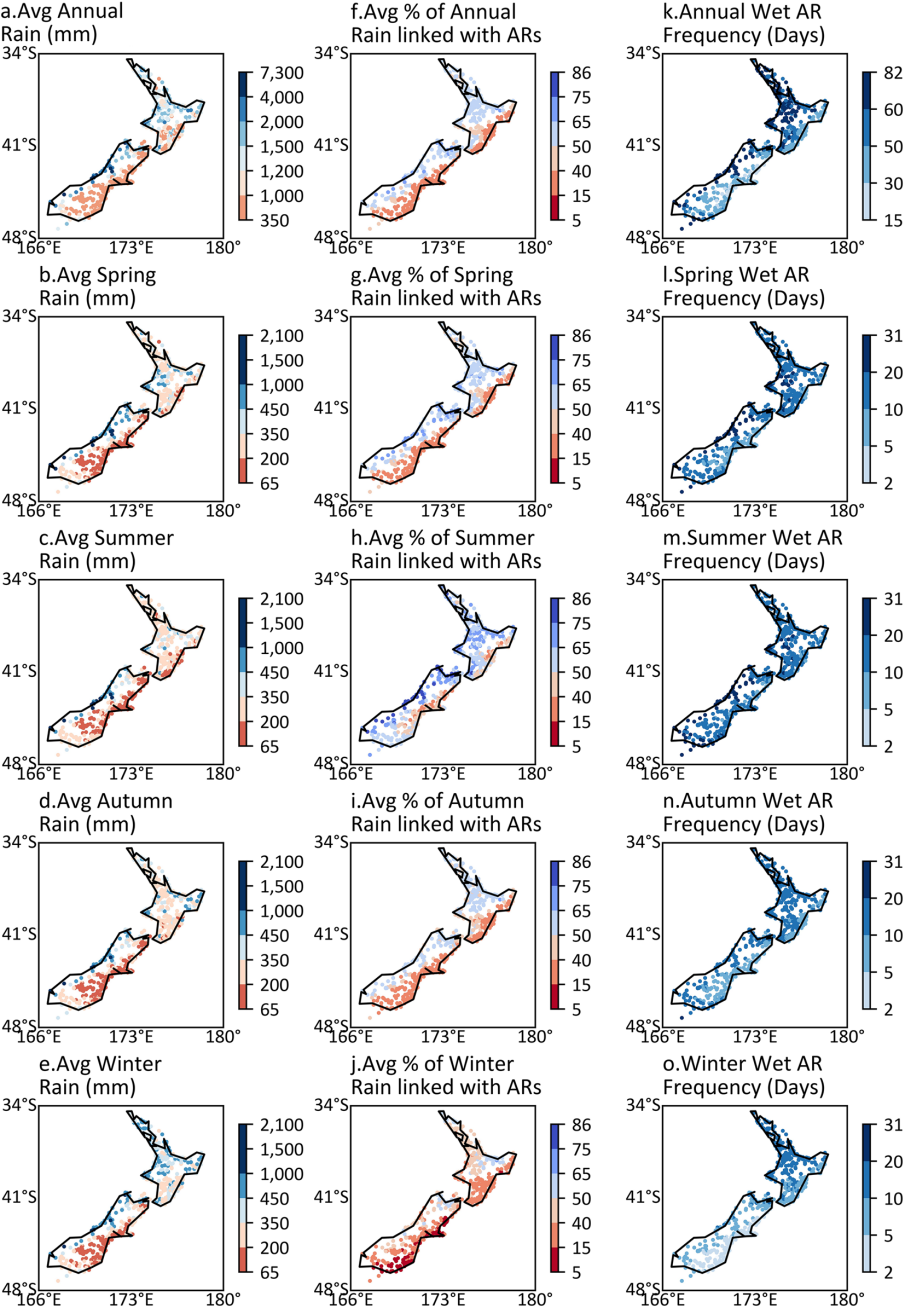
Maps of annual and seasonal rainfall totals, percentage of rain totals linked with ARs, and the frequency of wet AR days per year for each site. (**a**–**e**) Average annual and seasonal rainfall totals. (**f**–**j**) Annual and seasonal average percentages of rain totals linked with ARs. (**k**–**o**) Annual and seasonal frequency of wet AR days per year. Note that months of seasons are: Spring (SON), Summer (DJF), Autumn (MAM), Winter (JJA).

This section examines the impact of detected ERA-interim ARs (at a 0.125° × 0.125° horizontal grid resolution) on rainfall totals and the frequency of wet AR days across New Zealand. Average annual and seasonal rainfall totals are shown in Fig. 3a–e. Generally, higher rainfall totals occur on the western side of the mountain ranges and mountain peaks across New Zealand. The highest annual and seasonal rainfall totals are recorded on the west side of the mountain range in the South Island (the Southern Alps). Seasonality in rainfall totals is notable for the North Island, being highest in the winter and lowest in summer.

The average fraction of annual and seasonal rainfall totals linked with ARs for each rain gauge are shown in Fig. 3f–j. The frequency of wet AR days is shown in Fig. 3k–o. The annual and seasonal fraction of rainfall totals linked with ARs is mostly over 50% except during winter on the west side of mountain ranges, mountain peaks, and northern North Island. For these areas, the highest fractions are recorded in summer (75–86%) and the lowest recorded in winter (15–40%). The seasonal frequency of wet AR days in these areas ranges from 5 to 31 days, with the highest frequency in summer and lowest in winter (Fig. 3k–o).

### Impact of ARs on extreme rainfall events

**Figure 4:**
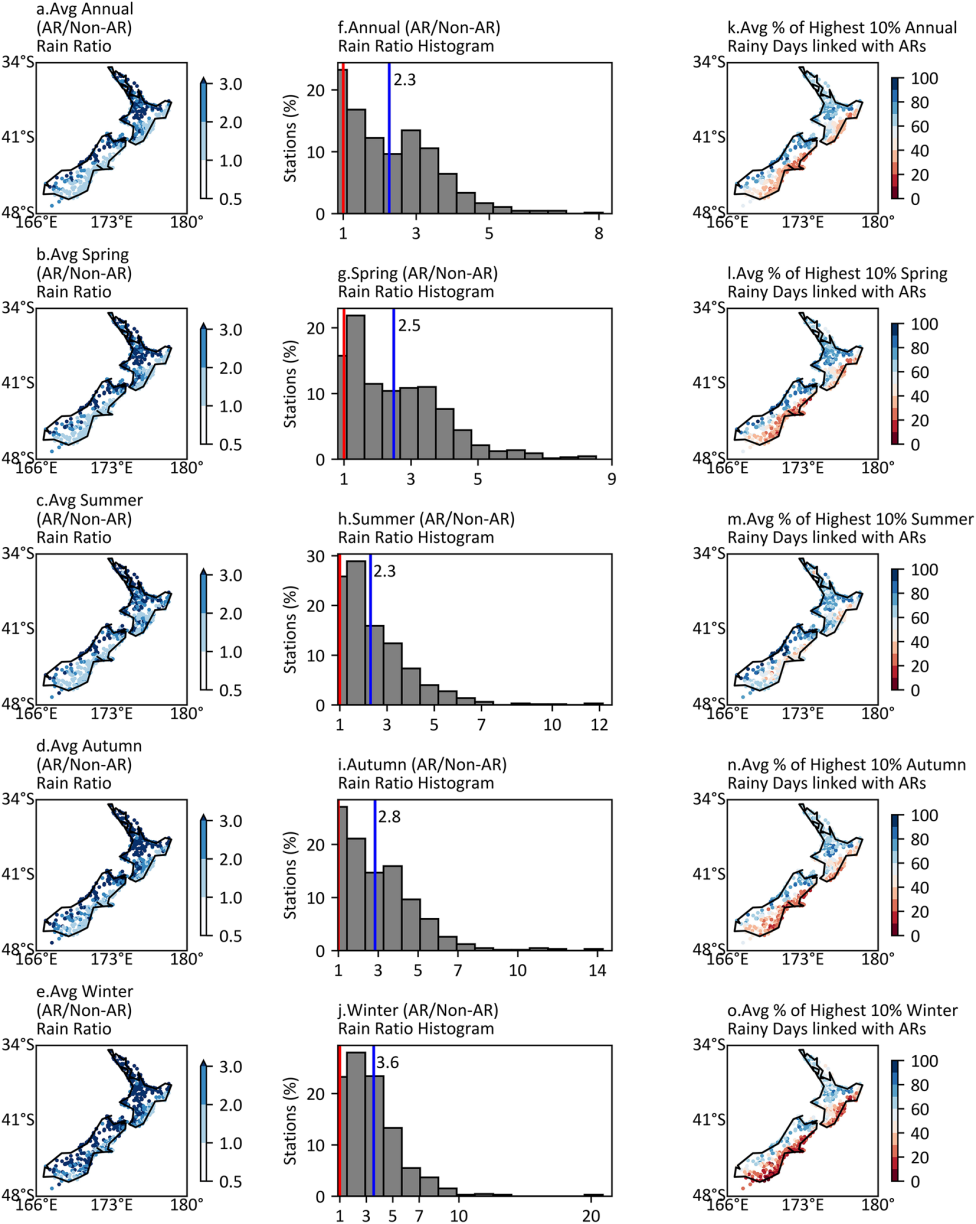
Maps of the average annual and seasonal AR/Non-AR rain ratio and the average percentage of the highest 10% rainy days linked with ARs, and histograms of AR/Non-AR rain ratio. (**a**–**e**) Annual and seasonal mean ratio of AR/Non-AR daily rainfall totals for each site. (**f**–**j**) Histograms of annual and seasonal mean ratio of AR/Non-AR daily rainfall totals. Red vertical lines in histograms locate the ratio 1, and blue vertical lines indicate the median values. (**k**–**o**) Annual and seasonal average percentage of the highest 10% rainy days linked with ARs. Note that months of seasons are: Spring (SON), Summer (DJF), Autumn (MAM), Winter (JJA).

The ratio of the median daily AR rainfall over non-AR rainfall at the annual and seasonal scales are shown in Fig. 4a–j. Interestingly, AR storms are found to produce greater than 2 times more daily rainfall than non-AR storms at most stations, and significantly greater than three times for the west side of mountainous areas and northern New Zealand. The ratios in winter are also notably high, particularly for some stations where the ratio can be over 20.

The connection of the most intense rainy days between ARs is explored by considering those days where rainfall exceeds the 90th percentile values of the series of daily rainfall. Figure 4k–o show the annual and seasonal fractions of those intense rainy days associated with ARs at each station. Generally, 50–98% of extreme rainfall events are associated with AR conditions on the west side of mountainous regions and northern New Zealand, depending on the season. The highest fraction for each station in these areas occurs in summer, whereby some exhibit values over 90% and 80% in the South and North Island, respectively.

## Discussion

The investigation of the impact of ARs on rainfall in this study is a major step toward understanding the hydrology and extreme rainfall events in New Zealand. In this study, the impact of ARs on rainfall was evaluated using a high-resolution atmospheric reanalysis dataset and a network of station rainfall data, combined with an automated AR detection algorithm introduced by Guan and Waliser^38^. Overall, ARs are found to have a strong hydrological impact over western areas of mountain ranges and mountain peaks, and northern New Zealand. Note that the discussion on the impact of ARs on rainfall only focuses on these areas.

New Zealand is an AR-highly impacted country^15,16,38^. The filamentary synoptic feature is notable whereby the median length of landfalling ARs is more than 5000 km (over 4700 km) for ECMWF products (other reanalysis datasets used), and the median length/width ratio is over 8 for all reanalysis datasets (Figs. 2 and S4–S6). However, it should be noted that coarser spatial resolution datasets tend to reduce the number of detected landfalling ARs. For example, coarsening the ERA-Interim and CFSR spatial resolution results in a notable reduction in the number of detected landfalling ARs (Table S1). This is also true for reanalysis datasets with coarser spatial resolution regardless of the reanalysis dataset (Table S1). Specifically, datasets with coarser spatial resolution might lead to the decreased occurrence in the comparably long landfalling ARs, because, for instance, the probability of landfalling ARs with length greater than 10,000 km in CFSR and MERRA-2 (coarser spatial resolution) is markedly less than in ERA-Interim and ERA-5 (higher spatial resolution) (Figs. 2a and S4–S6a). As a result, the median length of landfalling ARs for coarser spatial resolution is less than that of higher spatial resolution. The sensitivity analysis results in the geometric characteristics of landfalling ARs generally agree with Guan and Waliser^38^, as they found that coarsening the spatial resolution of the same reanalysis products can lead to a decreased occurrence of detected elongated ARs and shift the histogram of length/width ratio to left. Landfalling ARs generally have a northwest direction, but some exhibit a northeast direction along some coasts (Figs. 2e and S4–S6e). Characteristics in the landfalling AR direction across New Zealand generally agree with results in Guan and Waliser^38^. Additionally, landfalling ARs are generally more frequent and stronger in summer (DJF) than in winter (JJA) (Figs. 2c and S4–S6c, Table S2). Most landfall occurs along the west coasts through the country (Figs. 2e,f and S4–S6e,f), the frequency of landfall is relatively high over the central-eastern and northern parts of the North Island, and southern parts of the South Island (Figs. 2f and S4–S6f).

Overall, 50–65% of the annual and 40–86% of seasonal rainfall totals accumulated only within approximately 30–82 and 5–31 wet AR days respectively (Fig. 3), depending on the location of the rain gauge and season. This indicates that AR storms in these regions play a key role in water resources, and particularly true for the western coast of the Southern Alps where 50–65% of annual rainfall totals (nearly 7000 mm) accumulated within 60–82 wet AR days. Additionally, the highest AR contribution occurs in summer (DJF), which is also the season that the western coast of the Southern Alps receives the highest amount of rainfall and the season that landfalling AR occurrence and intensity is relatively high (Figs. 1, 2, 3 and S4–S6, Table S2). The results suggest that AR seasonality and orography largely determine the water availability in this region.

Rainfall in the country is strongly influenced by the interaction between the orography and the prevailing airflows^40,41^. As a result, the western areas of the country’s mountain ranges receive higher rainfall amounts^19,26^. North Island rainfall is relatively high in winter (JJA) as the frontal systems’ occurrence increases when the country is covered more by the westerly wind belt^37^. However, the belt moves further south during the summer (DJF) months and leads to lower rainfall in the North Island^19,37^. Consequently, the seasonal pattern of rainfall in the two islands differs^19^. Further study on the landfalling AR physical processes related to global circulation features and source of ARs around New Zealand is needed.

AR storms are more intense than non-AR storms all year round and found to produce 2–20 times the non-AR rainfall depending on the rain gauge location and season (Fig. 4). Particularly, cool-season daily rainfall intensity related to AR storms is more intensive compared with non-AR rain in most of New Zealand. The connection between the extreme rainfall events and ARs are notably high, ranging from 50% to 98% depending on the rain gauge locations and seasons (Fig. 4). Indeed, the percentage is over 90% in some areas on the west side of the Southern Alps in summer (DJF). As ARs are often related to fronts, the impact of ARs on extreme rainfall events in this study agrees with Catto and Pfahl^10^, although the 99th threshold was used in their study.

Further, results in this study suggest a potential connection between hydrological droughts and floods in northern New Zealand and where the orography dominates the weather progression in the country (Figs. 2f and S4–S6f). Droughts and floods are the most costly and damaging natural hazards in New Zealand^42^. Under the changing climate, the frequency and magnitude of floods are likely to increase as a result of the increased frequency and intensity of extreme rainfall in New Zealand^43^. Climate change is expected to also lead to increases in the frequency of droughts in vast areas of the North Island^44^, including regions where ARs are the major contributor to water resources. Moreover, climate change is suggested to result in a 60% and 20% increase in the AR frequency and strength in the southern midlatitudes, respectively^39^. Therefore, further work on investigating the connection between hydrological hazards and ARs focusing on regions exposed to both ARs and these hazards is needed for a better assessment of climate change impact on natural hazards.

## Methods

### Reanalysis and station rainfall data

It is common to employ more than one dataset to assess the robustness of AR detection techniques. Datasets used to identify ARs are the European Centre for Medium-Range Weather Forecasts (ECMWF) Interim (ERA-interim)^45^ and the most recent reanalysis dataset version ECMWF, namely the ERA-5 reanalysis project^46^. Atmospheric reanalysis data between 1979 and 2018 from ERA-interim and ERA-5 at 0.125° × 0.125° and 0.25° × 0.25° horizontal grid resolution were retrieved, respectively. Data used are 6-hourly specific humidity and zonal wind and meridional wind components at 20 vertical pressure levels (300 hPa to 1000 hPa), and land-sea mask. Additionally, detected ARs based on ERA-Interim, the Climate Forecast System Reanalysis^47^ (CFSR), and the Modern-Era Retrospective Analysis for Research and Applications^48^, Version 2 (MERRA-2) were retrieved from a global AR database^38^. Note that the AR detection period based on ERA-Interim, CFSR, and MERRA-2 is 1979–2018, 1979–2015 and 1980–2019, respectively, with a 6-hourly temporal resolution at 1.5° × 1.5°, 0.5° × 0.5°(and 1.5° × 1.5°), and 0.5° × 0.625° horizontal grid resolution, respectively. Table S1 summarised all the information about the datasets employed. The integrated water vapour transport (IVT) intensity at each grid cell was calculated in an Eulerian framework^22,25,49^ as:

1 $$IVT= \sqrt{{IVT}_{x}^{2}+{IVT}_{y}^{2}}$$

2 $${IVT}_{x}=\frac{1}{g}{\int }_{1000hPa}^{300hPa}qu \, dp$$

3 $${IVT}_{y}=\frac{1}{g}{\int }_{1000hPa}^{300hPa}qv \, dp$$

where *q*, $u$, and $v$ are specific humidity (kg kg^-1^), zonal and meridional vectors (m s^-1^), respectively; *g* is the gravitational acceleration (9.81 m s^−2^), and *dp* (Pa) is the pressure difference between two adjacent atmospheric pressure levels. Note that all parameters in Eqs. (2) and (3) were collected at discrete atmospheric pressure levels from the selected datasets. According to Guan and Waliser^38^, the median length of global 1997–2014 ARs is about 3,665.1 km. For the ARs detected in the Southern Hemisphere, ARs commonly have a northwest direction with a median degree of 120.6°, and the median latitude of the AR equatorward end, centroid, and the poleward end is 28.5°S, 41.1°S, and 49.5°S respectively. Considering that the longitude length of a degree at 49.5°S is approximately 72 km and the first eastward meridian line across New Zealand is at about 166°E. Therefore, data retrieved over the 0–70°S and 100°E–120°W domain is considered adequate (166°E–100°E = 66°, 66° × 72 km/° = 4725 km > 3665.1 km).

**Figure 5:**
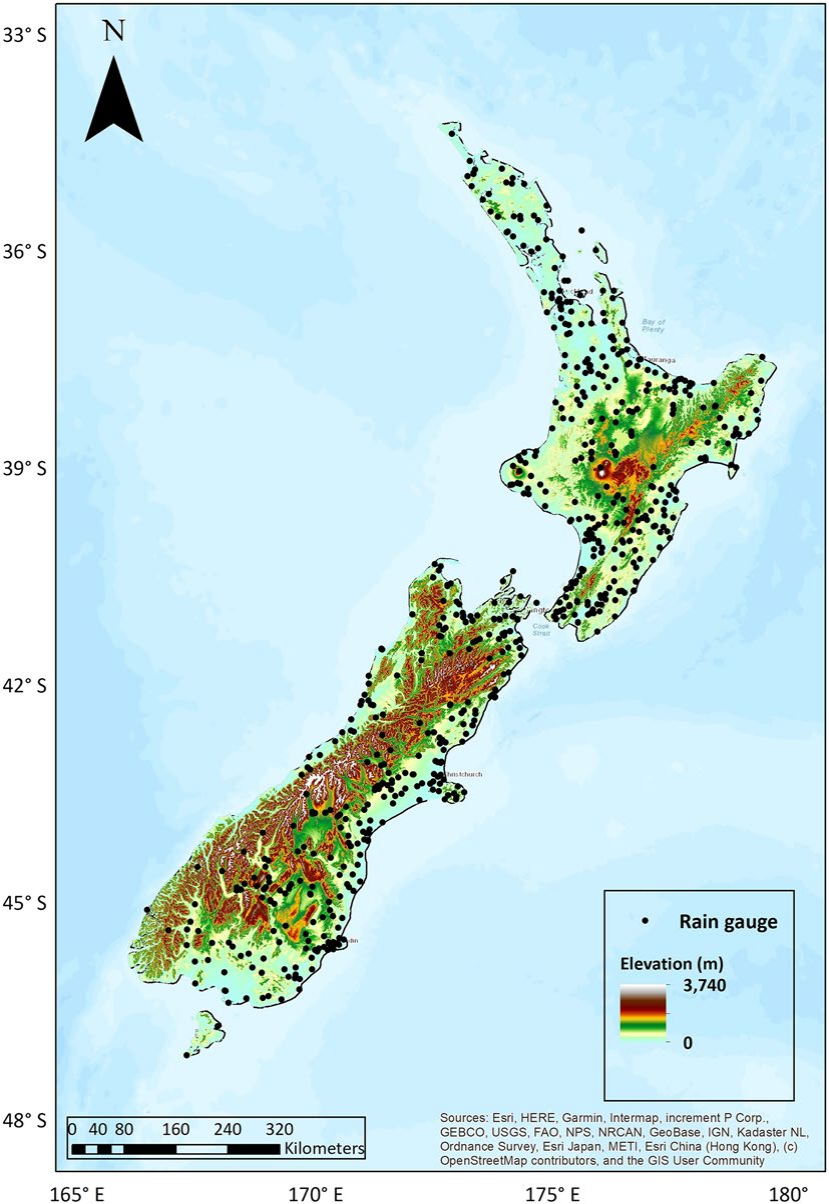
A map of New Zealand showing the location of 654 rain gauges.

Station-based daily rainfall totals were obtained from New Zealand’s national climate database web system hosted by the National Institute of Water and Atmospheric Research (NIWA). Note that the periods of records differ among sites, and the observation times of the day are 2100 UTC (2000UTC) during the wintertime (summertime). Sites with less than 10 years of daily rainfall records between (September–August) 1979 to 2018 and completeness less than 100% were excluded because the lack of daily rainfall data could potentially lead to under- or overestimations in the hydrological impacts of ARs. Given these conditions, a total of 654 stations with the minimum, maximum, and mean length of 10, 39, and 19 years respectively were selected over the whole country. Figure 5 shows rain gauge locations.

### AR detection method

**Figure 6:**
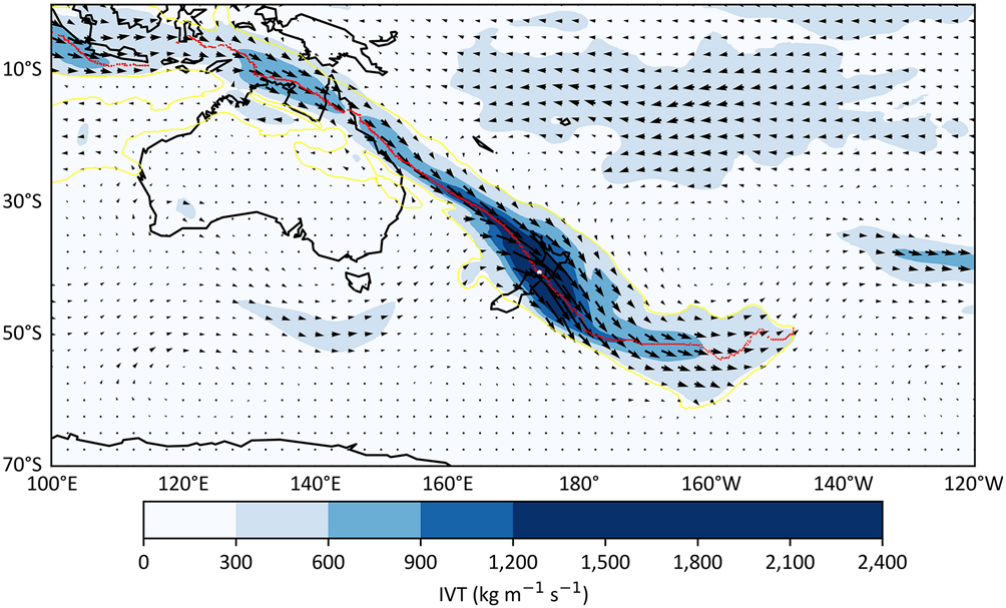
An example of detected AR by the detection algorithm at UTC 2010-12-27 18:00:00. The outputs of the algorithm include the shape boundary (yellow), axis (red), landfall location (white). Colour shading area and vectors refer to the AR IVT magnitude (kg m^−1^ s^−1^) and direction, respectively. This case was one of the strongest ARs between 1979 and 2018. The landfall location is at (174°E, 40.625°S) with the IVT magnitude of 2176 kg m^−1^ s^−1^ and 145° direction. The mean AR IVT magnitude and direction is 397 kg m^−1^ s^−1^ and 123°, respectively. The length and width is 13,236 km and 1216 km, respectively.

CFSR and MERRA-2 ARs were obtained from a global AR database^38^. ERA-interim and ERA-5 ARs were detected based on the AR detection algorithm introduced in Guan and Waliser^38^. In the algorithm, IVT and IVT threshold (monthly 85th percentile within a 5-month window centred at that month) for each grid cell and the land-sea mask from the two datasets are the inputs. Note that the land-sea mask values were adjusted only to contain grid cells represent New Zealand landmasses (1 s represent New Zealand landmasses, 0 s refer to others). Briefly, criteria of the AR detection algorithm include the IVT intensity at each grid cell within a contiguous region being above the IVT threshold and a fixed limit (IVT > 100 kg m^−1^ s^−1^) for that grid cell, the appreciable poleward direction of the AR (IVT_y_ > 50 kg m^−1^ s^−1^), and the length of an AR greater than 2000 km with a length/width ratio greater or equal to 2. Outputs from the algorithm include the variables of shape, axis, and landfall location of the ARs in NetCDF files and detailed characteristics of landfalling ARs in text files. The AR shape variable was used to compute the AR area, the axis was used to calculate the AR length, and the width was calculated as the area divided by the length. The landfall location was marked as the first grid cell that the AR axis intersects the coastlines alone the IVT direction and that AR was labelled as a landfalling AR. The detailed description of the AR detection procedure and criteria of this technique are in Guan and Waliser^38^. Figure 6 shows one of the strongest detected ARs passing New Zealand.

### AR frequency and AR impacts on rainfall

The AR frequency was calculated as the average number of days per year that an AR was detected for each grid cell. The calculation was based on the shape of detected ARs. For example, at least one of the four reanalysis time steps per day meeting the AR detection criteria for it to be counted as an AR day^21^. Seasonal AR frequency is expressed as the anomalies from the mean seasonal AR days. The coefficients of variation (CV) for annual and seasonal AR days were calculated to evaluate the interannual variation of AR frequency. The mean monthly percentage of landfalling AR occurrence was computed as the mean of the number of landfalling ARs detected for that month over each year within the period of each dataset.

Consider a given station with daily rainfall records (measured at 2000 UTC or 2100 UTC) on 15 June 2000. To determine if the rainfall should be associated with an AR, the first step is to search for AR conditions (based on AR shape) at the four grid points that enclosed the station site between 2000 UTC (2100 UTC) 14 June 2000 and 2000 UTC (2100 UTC) 15 June 2000. If any of the four ERA-interim time steps that AR conditions (based on AR shape) were found in the 4 grid points, the daily rainfall (> 0 mm) recorded between this period is considered to be AR-generated rainfall, and that day herein considered as a wet AR day. Unlike previous studies that any of the four grid points that AR conditions were present^21^ and rainfall in the next day^17,21,50^ (e.g., here the 16 June 2000, if any) considered as AR-precipitation event, only AR conditions presented at 4 grid points and rainfall occurred in the same day were considered as AR-generated rainfall in the present study.

Due to positive skewness in the daily rainfall data (not shown), daily rainfall intensity under AR and non-AR conditions were compared by the median daily rainfall^21^ (calculated separately for days with and without AR conditions at each station, and the ratio was subsequently obtained). Further, the fraction of annual and seasonal highest 10% rainfall events that are attributed to ARs were also investigated.

## Supplementary Information

Supplementary Information.

## Data Availability

Data used in this study were obtained freely online: ERA-Interim: https://apps.ecmwf.int/datasets/data/interim-full-daily. ERA-5: https://cds.climate.copernicus.eu/cdsapp#!/dataset/reanalysis-era5-pressure-levels?tab=overview. CFSR: https://rda.ucar.edu/. MERRA-2: https://gmao.gsfc.nasa.gov/reanalysis/MERRA-2/. Daily rainfall: https://cliflo.niwa.co.nz/.

## Code availability

The Python code and Excel VBA code used to compute AR frequency and quantify AR impacts on rainfall are available from the corresponding author upon request.
