# Supplementary material for: The impact of atmospheric rivers on rainfall in New Zealand
Source: Sci Rep. 2021 Mar 12;11:5869. doi: 10.1038/s41598-021-85297-0 (PMC7955079; doi:10.1038/s41598-021-85297-0)
Supplement: Supplementary file 1 — Supplementary Information. [file 41598_2021_85297_MOESM1_ESM.pdf]

SUPPLEMENTARY INFORMATION FOR

The Impact of Atmospheric Rivers on Rainfall in New Zealand

Jingxiang Shu[1*], Asaad Y. Shamseldin[1+], Evan Weller[2+]

[1]Department of Civil and Environmental Engineering, The University of Auckland, Private Bag 92019, Auckland Mail Centre, Auckland 1142, New Zealand

[2]School of Environment, The University of Auckland, Private Bag 92019, Auckland Mail Centre, Auckland 1142, New Zealand

[*]corresponding. jshu987@aucklanduni.ac.nz

Table S1. A detailed description of the employed reanalysis datasets and the number of detected landfalling ARs for each dataset

Dataset	Period	Spatial resolution	Temporal resolution	No. of detected Landfalling ARs
ERA-interim	Sep 1979 - Aug 2018	0.125°×0.125°	6-hourly	17,876
ERA-interim	Jan 1979 - Aug 2019	1.5°×1.5°		4,057
ERA-5	Sep 1979 - Aug 2018	0.25°×0.25°		17,997
CFSR	Jan 1979 - Dec 2015	0.5°×0.5°		12,904
CFSR	Jan 1979 - Dec 2015	1.5°×1.5°		3,445
MERRA-2	Jan 1980 - Dec 2019	0.5°×0.625°		14,715

Table S2. Mean percentage of the occurrence of detected landfalling ARs for each month for each dataset

Dataset (spatial resolution)	Sep	Oct	Nov	Dec	Jan	Feb	Mar	Apr	May	Jun	Jul	Aug
ERA-interim (0.125°×0.125°)	8%	11%	11%	12%	12%	9%	8%	7%	7%	6%	5%	6%
ERA-5 (0.25°×0.25°)	8%	11%	11%	12%	13%	10%	8%	7%	6%	6%	5%	6%
CFSR (0.5°×0.5°)	8%	10%	9%	10%	10%	8%	7%	8%	8%	8%	6%	7%
CFSR (1.5°×1.5°)	8%	12%	11%	10%	10%	8%	7%	7%	9%	8%	5%	6%
MERRA-2 (0.5°×0.625°)	8%	10%	9%	10%	10%	8%	8%	8%	8%	8%	7%	8%
ERA-interim (1.5°×1.5°)	8%	12%	11%	9%	10%	10%	7%	8%	9%	9%	6%	7%
Mean	8%	11%	10%	10%	11%	9%	8%	7%	8%	7%	6%	7%

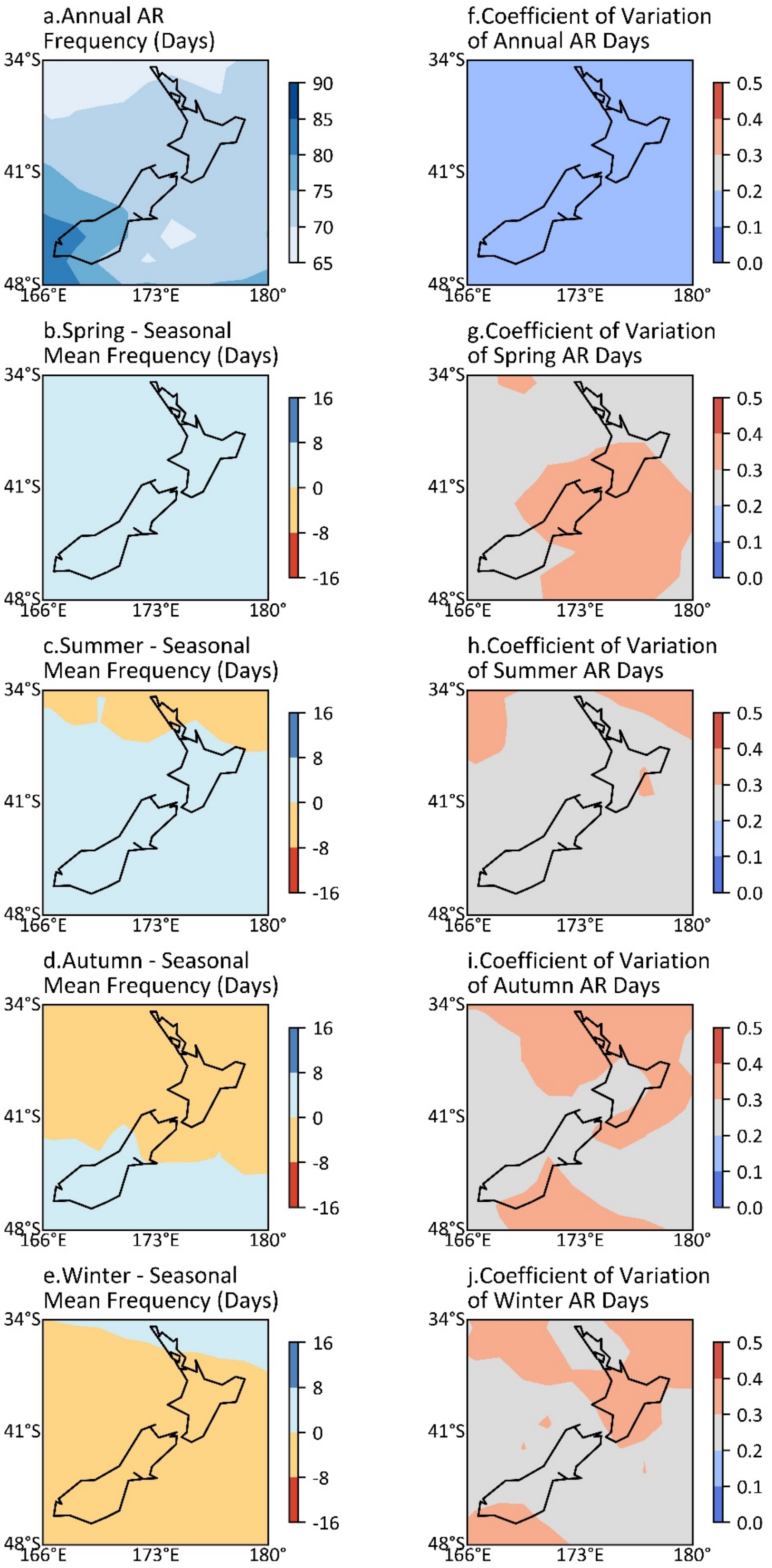

Figure S1. The climatology of AR frequency over New Zealand (ERA-interim at 1.5°×1.5° grids). **a** Annual frequency of AR days on each grid cell per year. **b-e** Difference between seasonal AR frequency and its seasonal mean frequency on each grid cell per year. **f-j** Annual and seasonal coefficients of variation of AR frequency for each grid cell. Note that months of seasons are: Spring (SON), Summer (DJF), Autumn (MAM), Winter (JJA).

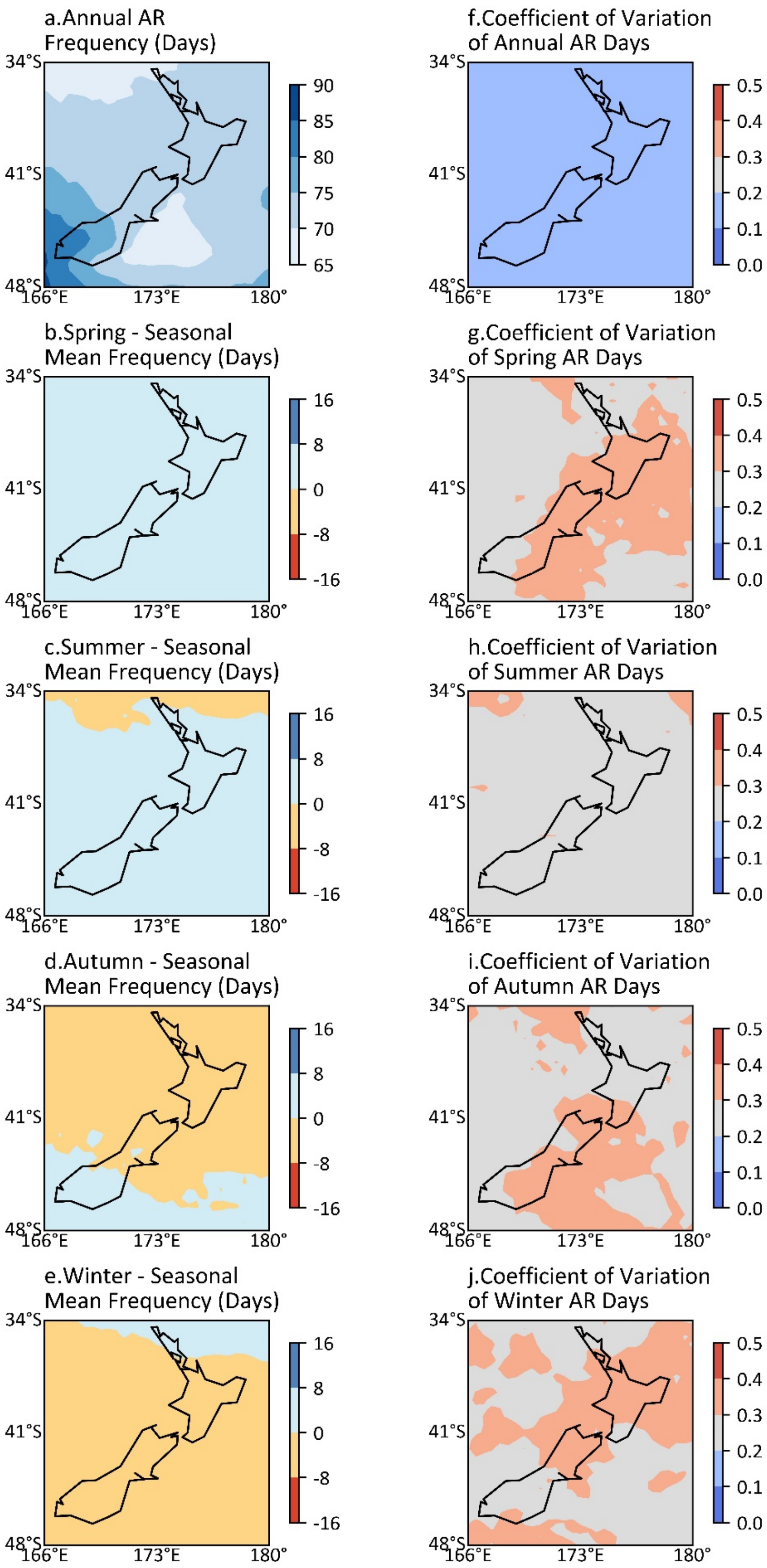

Figure S2. The climatology of AR frequency over New Zealand (CFSR at 0.5°×0.5° grids). **a** Annual frequency of AR days on each grid cell per year. **b-e** Difference between seasonal AR frequency and its seasonal mean frequency on each grid cell per year. **f-j** Annual and seasonal coefficients of variation of AR frequency for each grid cell. Note that months of seasons are: Spring (SON), Summer (DJF), Autumn (MAM), Winter (JJA).

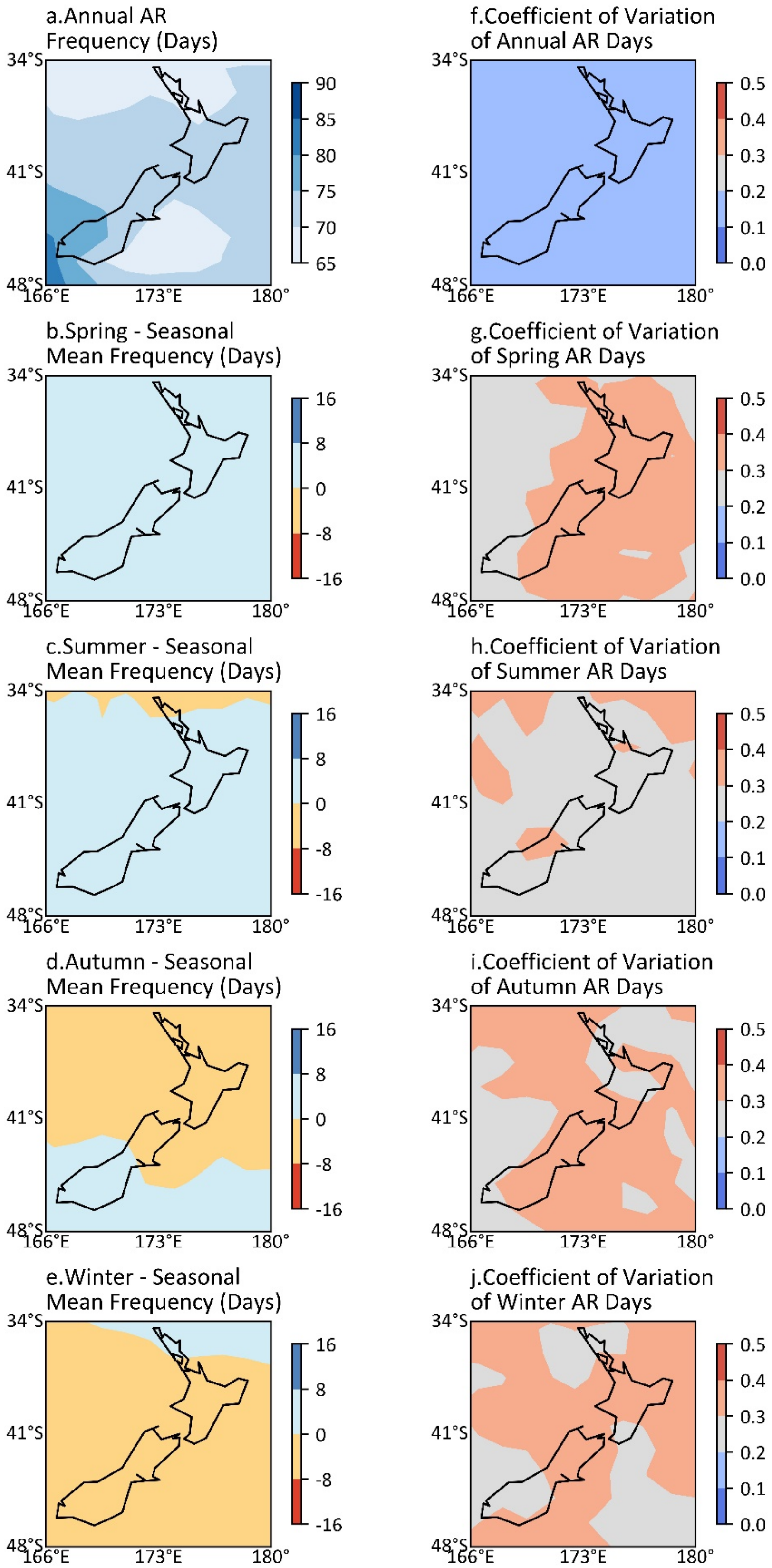

Figure S3. The climatology of AR frequency over New Zealand (CFSR at 1.5°×1.5° grids). **a** Annual frequency of AR days on each grid cell per year. **b-e** Difference between seasonal AR frequency and its seasonal mean frequency on each grid cell per year. **f-j** Annual and seasonal coefficients of variation of AR frequency for each grid cell. Note that months of seasons are: Spring (SON), Summer (DJF), Autumn (MAM), Winter (JJA).

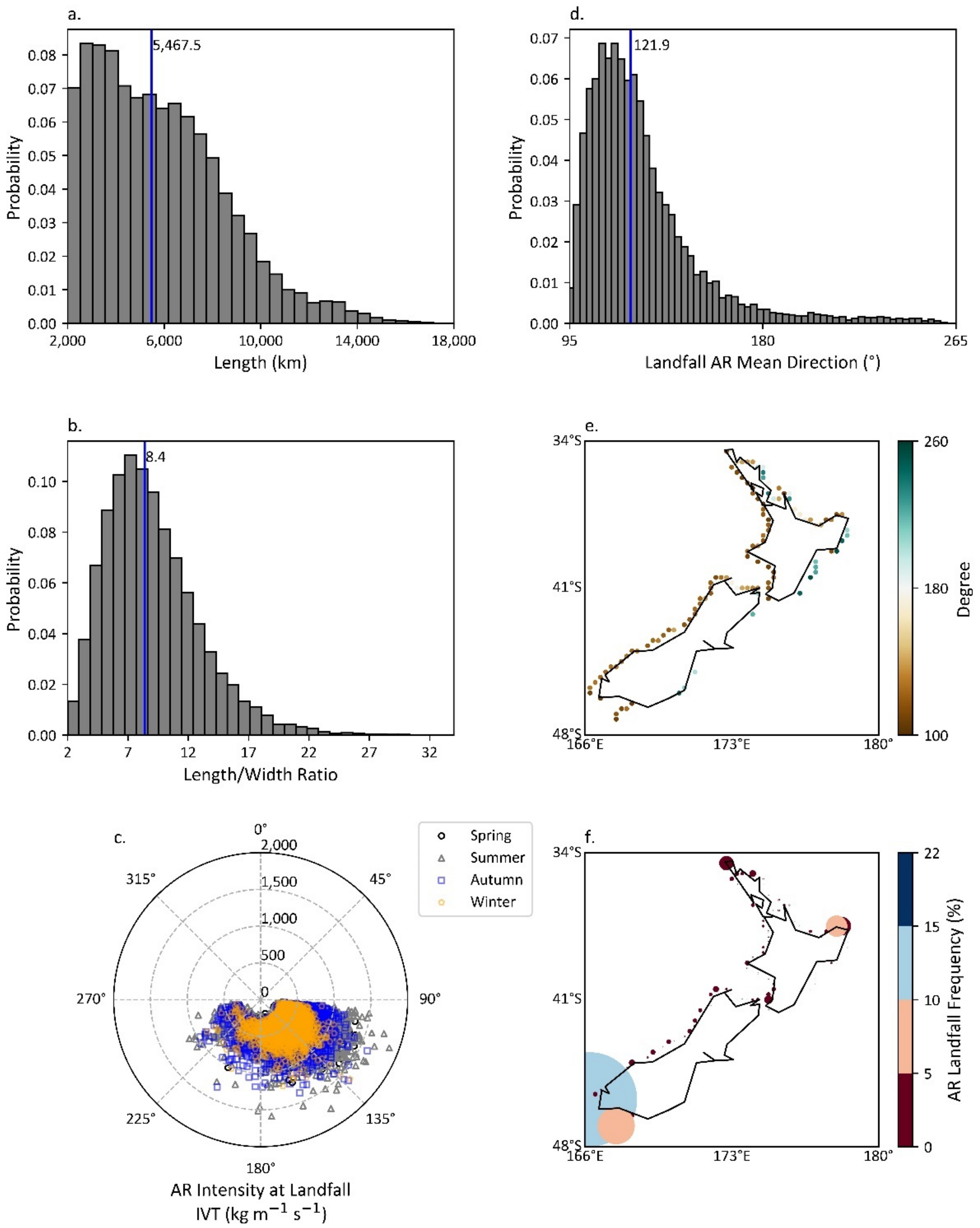

Figure S4. Histograms of detected 1979-2018 ERA-5 (0.25°×0.25°) landfalling ARs' length (**a**), Length/width ratio (**b**), Landfalling AR mean direction (**d**), the blue vertical lines indicate the median value in each histogram. **c** Mean landfalling AR direction (angular coordinate) and seasonal landfalling AR intensity (radial coordinate) at landfall. **e** Landfalling ARs mean direction at landfall. **f** Proportional map of AR landfall frequency at landfall. Note that months of seasons are: Spring (SON), Summer (DJF), Autumn (MAM), Winter (JJA).

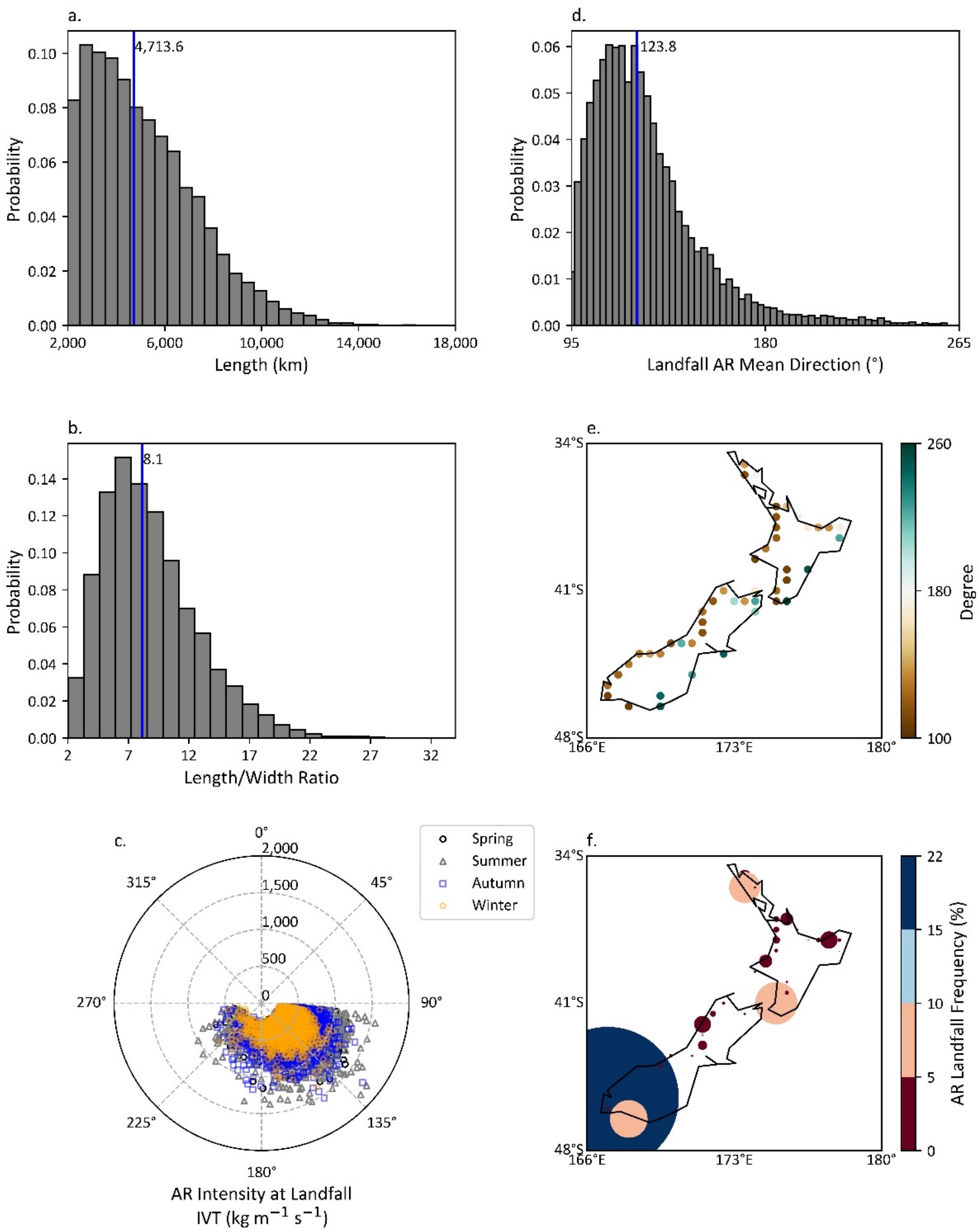

Figure. S5 Histograms of detected 1979-2015 CFSR (0.5°×0.5°) landfalling ARs' length (**a**), Length/width ratio (**b**), Landfalling AR mean direction (**d**), the blue vertical lines indicate the median value in each histogram. **c** Mean landfalling AR direction (angular coordinate) and seasonal landfalling AR intensity (radial coordinate) at landfall. **e** Landfalling ARs mean direction at landfall. **f** Proportional map of AR landfall frequency at landfall. Note that months of seasons are: Spring (SON), Summer (DJF), Autumn (MAM), Winter (JJA).

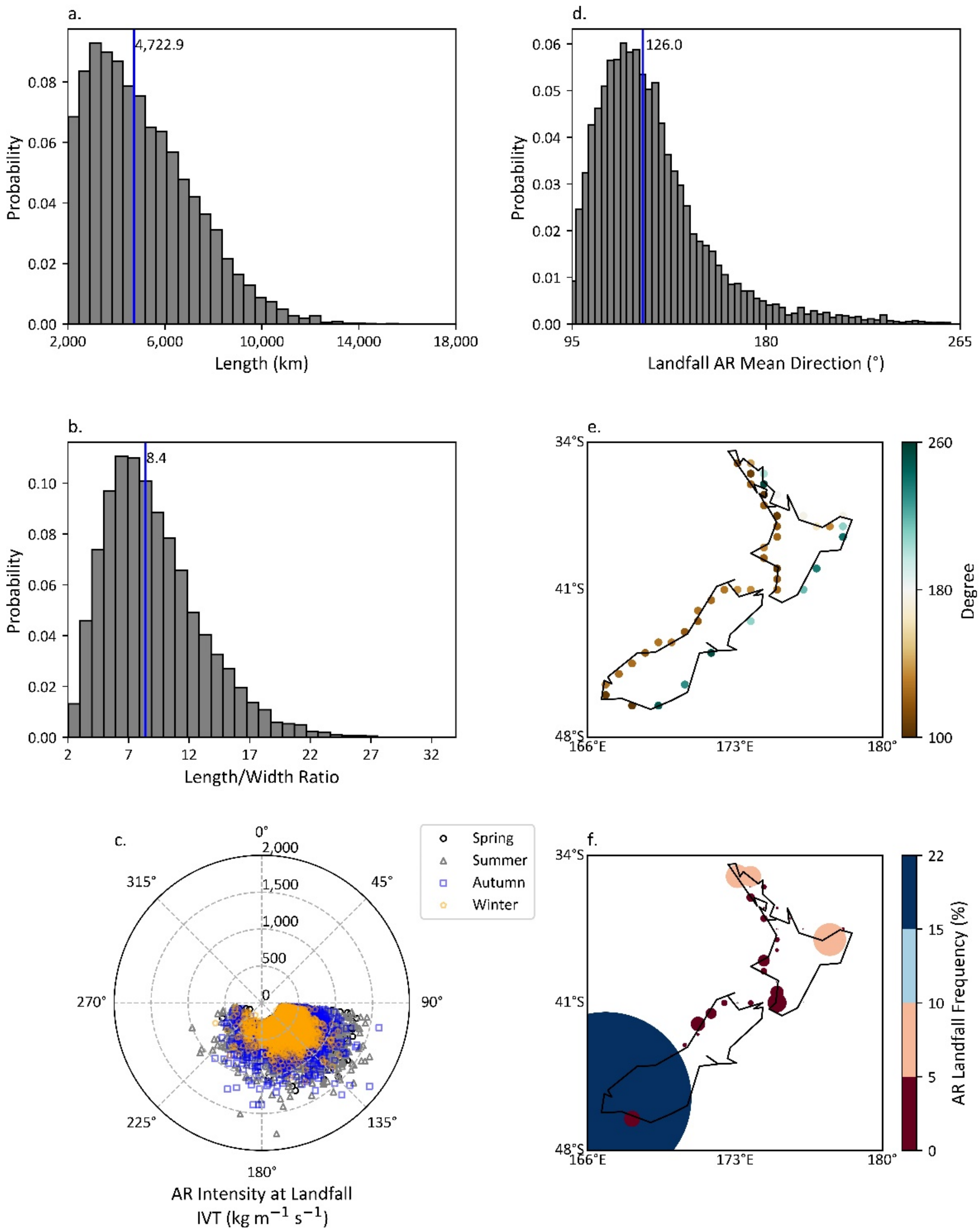

Figure. S6 Histograms of detected 1980-2019 MERRA-2 (0.5°×0.625°) landfalling ARs' length (**a**), Length/width ratio (**b**), Landfalling AR mean direction (**d**), the blue vertical lines indicate the median value in each histogram. **c** Mean landfalling AR direction (angular coordinate) and seasonal landfalling AR intensity (radial coordinate) at landfall. **e** Landfalling ARs mean direction at landfall. **f** Proportional map of AR landfall frequency at landfall. Note that months of seasons are: Spring (SON), Summer (DJF), Autumn (MAM), Winter (JJA).
